# Phenotypic insecticide resistance in arbovirus mosquito vectors in Catalonia and its capital Barcelona (Spain)

**DOI:** 10.1371/journal.pone.0217860

**Published:** 2019-07-05

**Authors:** Krijn Paaijmans, Marco Brustollin, Carles Aranda, Roger Eritja, Sandra Talavera, Nonito Pagès, Silvie Huijben

**Affiliations:** 1 ISGlobal, Barcelona, Spain; 2 School of Life Sciences, Center for Evolution and Medicine, Arizona State University, Tempe, AZ, United States of America; 3 The Biodesign Center for Immunotherapy, Vaccines and Virotherapy, Arizona State University, Tempe, AZ, United States of America; 4 Centre de Recerca en Sanitat Animal (CReSA IRTA), Barcelona, Spain; 5 The Center for Infectious Disease Dynamics, and the Huck Institutes of The Life Sciences, Millennium Science Complex, Pennsylvania State University, University Park, PA, United States of America; 6 Servei de Control de Mosquits, Consell Comarcal del Baix Llobregat, Barcelona, Spain; 7 CREAF, Cerdanyola del Vallès, Spain; 8 CIRAD, UMR ASTRE, Petit Bourg, Guadeloupe, France; 9 ASTRE, CIRAD, INRA, Montpellier University, Montpellier, France; Institut de recherche pour le developpement, FRANCE

## Abstract

A range of mosquito species that belong to the Culicidae family are responsible for the worldwide transmission of infectious arboviral diseases such as dengue fever, Zika, West Nile fever and Chikungunya fever. Spain is at risk of arbovirus outbreaks, as various arboviral diseases are frequently introduced and it has established competent vector populations. Autochthonous human cases of West Nile virus have been reported infrequently since 2004, and since October 2018 three autochthonous human case of dengue fever have been confirmed. In response to an outbreak of any arboviral disease, space spraying or fogging will be implemented to control adult mosquito populations. To ensure adulticiding is cost-effective, the insecticide susceptibility status of vectors throughout Catalonia, an autonomous region in north-eastern Spain, was assessed through standardized WHO tube and CDC bottle bioassays. All *Culex pipiens* populations tested were resistant to at least one of the pyrethroids tested, whereas *Aedes albopictus* populations were susceptible to all pyrethroids tested. More detailed studies on the *Cx*. *pipiens* populations from the Barcelona area (the capital and largest city of Catalonia) revealed resistance to all four classes of public health insecticides available (pyrethroids, carbamates, organophosphates and organochlorides). All *Ae*. *albopictus* populations were susceptible to those classes, except for one of the tests performed with pirimiphos-methyl (an organophosphate). Pyrethroids are currently the first line chemical class to be used in space spray operations in response to an outbreak of an arboviral disease. While pyrethroids can be effective in reducing *Ae*. *albopictus* populations, this class may not be effective to control *Cx*. *pipiens* populations.

## Introduction

A range of mosquito species that belong to the Culicidae family are responsible for the worldwide transmission of infectious arboviral diseases such as dengue fever, Zika, West Nile fever and Chikungunya fever. On the European continent, autochthonous disease cases (i.e. infections acquired by mosquito transmission in a currently non-endemic area) have been observed in several Mediterranean countries. Autochthonous transmission of dengue virus (DENV) has occurred in France in 2010 [1], Croatia in 2010 [2] and on the Portuguese archipelago Madeira in 2012 [3], with more than 2000 local cases reported in the latter. Autochthonous West Nile virus (WNV) cases have been seen in France [4, 5] and Italy in 2011 [6] and the same countries experienced outbreaks of Chikungunya virus (CHIKV), Italy in 2007 [7] and 2017 [8] and France in 2010 [9] and 2017 [10].

In Spain, few clinical cases of WNV have been observed in 2004, 2010 and 2016 [11–13] and serological evidence confirms its presence in Catalonia [14]. Mosquitoes and birds maintain the WNV enzootic transmission cycle [15] and periodic cases have been identified in horses since 2010 [16]. Very recent (October 2018), three cases of autochthonous dengue have been confirmed in Spain [17], and the virus has been detected in its local vector *Aedes albopictus* [18]. This outbreak was arguably expected given that key ingredients for transmission were present. First, pathogens such as DENV, Zika virus (ZIKV) and CHIKV are frequently imported by travelers [19–21]. Second, arboviruses such as DENV, ZIKV, CHIKV and Yellow Fever virus (YFV) can be transmitted by *Ae*. *albopictus* (the Asian tiger mosquito), which is abundant across the Mediterranean coast [22]. In Spain, this species was first detected in the Barcelona area (Sant Cugat del Vallès) in 2004 [23], but is slowly spreading across the Spanish mainland [24]. *Ae*. *aegypti*, another -typically more competent- vector of the same arboviruses, has recently been detected on the Canary Islands [25]. Other viruses such as WNV and Rift Valley fever virus (RVFV) can be transmitted by *Ae*. *albopictus*, *Aedes caspius* and several *Culex* species, including *Cx*. *pipiens* s.l. and *Cx*. *theileri* [26, 27]. And *Cx*. *pipiens* was also found to be infected with Usutu virus (USUV) in Catalonia [28]. All these aforementioned mosquito vectors are abundant in Spain [29, 30], although their role as vector has not been clearly established for some viruses yet.

As there is no specific prophylactic treatment for most of these arboviruses, disease prevention and control are currently limited to vector control interventions. Interventions in Spain include (i) adulticiding (space spraying by ground, also known as fogging), (ii) larviciding, and (iii) larval source reduction via public cooperation and cleaning of landfills [31]. Larviciding is currently used in different mosquito habitats such as flooded areas, agricultural areas and urban sites during mosquito season, usually between early spring and autumn, with biological insecticides, mainly *Bacillus thuringiensis* var. *israelensis* (Bti) products. Occasionally ground fogging is implemented with different pyrethroids, depending on mosquito density populations and risk of vector transmission, but adulticide use is infrequent. Main regions where mosquito activity is important are the Baix Llobregat in Barcelona, the Ebro delta in Tarragona and the Roses Bay in Girona. Also, many municipalities such as Barcelona city conduct mosquito control operations. In emergency situations, such as sudden disease outbreaks, the application of adulticides (fogging on the ground with pyrethroids) is the planned intervention to rapidly target local vector populations [32]. To ensure this intervention is cost-effective, an insecticide needs to be selected to which the vectors are susceptible. As such, the level of insecticide susceptibility in a range of competent vectors needs to be monitored regularly, to allow for quick and effective decision making. Here we present data on the insecticide susceptibility in various potential arbovirus vectors (*Cx*. *pipiens*, *Ae*. *albopictus* and *Ae*.*caspius*) in Catalonia.

## Materials and methods

This paper combines different insecticide resistance datasets that have been collected in different geographical regions by different research teams for different mosquito species over the past 5 years. The methodology and results sections are presented per mosquito species for clarity.

### Study area

**Fig 1:**
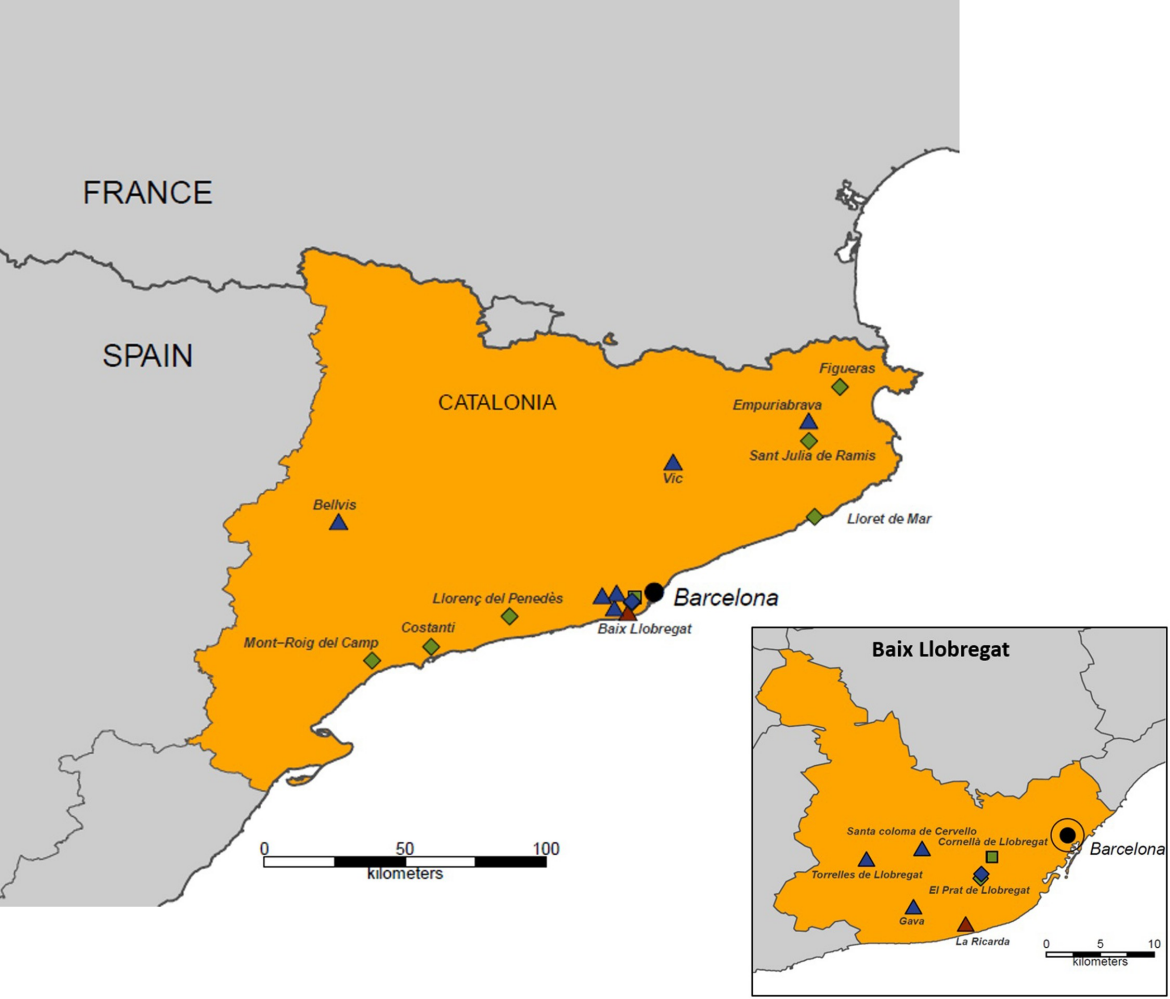
Map of Catalonia (Spain) highlighting the areas where *Aedes albopictus* (green symbols; 2012–2014, 2016), *Culex pipiens* (blue symbols; 2012–2014, 2015, 2016, 2017) and *Aedes caspius* (red symbol; 2017) were collected for the insecticide susceptibility assays. Diamonds indicate mosquitoes collected as eggs, triangles show sites with immature collections, and squares indicate adult collections.

The potential arbovirus vectors *Cx*. *pipiens*, *Ae*. *albopictus* and *Ae*.*caspius* were collected between 2012 and 2017 in Catalonia, an autonomous region in north-eastern Spain along the Mediterranean coast (Fig 1, drawn using public Global Administrative Areas (GADM) data [33] in the base package of R [34]). The region is about 32,000 km^2^ large, contains approximately 7.5 million inhabitants and has a coastal and inland Mediterranean climate. Subsequent tests focused on the Baix Llobregat region in 2015 to 2017, an area in close proximity to Barcelona, the capital and largest city of Catalonia. The Llobregat delta is one of Catalonia's most important wetland zones, and the park is mainly used for recreation. In addition, there are large agricultural areas and over 800,000 inhabitants living in several towns. The area receives a high number of international visitors every year with the international airport Barcelona-El Prat situated in the Baix Llobregat study area which handled over 44 million passengers in 2016 [35]. Of the 54 mosquito species found in Spain [30], including the invasive *Ae*. *albopictus* [23], 47 have been detected in Catalonia and 18 in the Baix Llobregat region [36]. Of these, *Cx*. *pipiens*, *Ae*. *albopictus* and *Ae*. *caspius* are the species that have most negative impact in the human population, not only for their ability to transmit human pathogens but also for their feeding habits. Aggressive species like *Ae*. *albopictus* and *Ae*. *caspius* can decrease human quality of life in affected communities by reducing the time people can spend outdoors [37] and/or increasing the need for preventive measures (i.e. repellents or nets).

No specific permissions were required for mosquito sampling in these locations, and sampling was carried out under the supervision of public health authorities of the Generalitat of Catalonia. Field studies did not involve endangered or protected species.

### Mosquito collections and rearing

#### Culex pipiens

Immature stages of *Culex pipiens* were collected from various geographical locations in Catalonia (Fig 1) between 2012 and 2014 and reared to adults in the laboratory on a TetraMin Tropical fish flakes diet at 23°C, 80%RH. Emerging mosquitoes had *ad libitum* access to 10% sucrose solution. To identify the correct taxonomic biotype (*Cx*. *pipiens pipiens* biotype *pipiens*, *Cx*. *pipiens pipiens* biotype *molestus* and *hybrid* between *pipiens* and *molestus* biotype) 10 specimens for each locality were selected and analysed by PCR [38]. In 2015 and 2016, immature stages of *Cx*. *pipiens* were collected from Torrelles de Llobregat (Fig 1) and reared to adult mosquitoes on a diet of Frippak 3CD fish food (Baasrode, Belgium) at 25°C and >60% RH. Emerging mosquitoes had *ad libitum* access to 10% honey-water solution. In 2017, egg rafts of *Cx*. *pipiens* were collected at el Prat de Llobregat (Fig 1). Eggs were allowed to hatch and the larvae were reared to adult in the laboratory under the same conditions as in 2015 and 2016. Taxonomic biotype was not assessed for the 2015–2017 mosquito specimens.

#### Aedes albopictus

Eggs of *Ae albopictus* were collected between 2012 and 2014 throughout Catalonia (Fig 1) using oviposition traps (ovitraps) consisting in black plastic 5L bucket half filled with water. A permeable wooden stick made of high-density fibreboard was added as oviposition medium. Eggs were stored under dry conditions and incubated with dechlorinated tap water until L1 larvae emerged. Larvae were maintained on a TetraMin Tropical fish flakes diet at 23°C, 80%RH. In 2016, adult *Ae*. *albopictus* were collected in Cornellà de Llobregat (Fig 1) using BG-Sentinel traps (Biogents AG, Regensburg, Germany) equipped with CO_2_ (dry ice) and a BG-Lure cartridge. Adult mosquitoes were provided 10% honey water *ad libitum* until the susceptibility test (6 days post-collection) at 25°C and >60% RH. For a second set of susceptibility tests in 2016, *Ae*. *albopictus* eggs were collected using ovitraps in a rural area in el Prat de Llobregat (Fig 1). Eggs were stored under dry conditions and incubated with dechlorinated tap water until L1 larvae emerged. Emerged larvae were reared to adult mosquitoes on a diet of Frippak 3CD fish food at 25°C and >60% RH.

#### Aedes caspius

A period of heavy rainfall allowed us to collect fourth instar larvae and pupae of the floodwater mosquito *Ae*. *caspius* at La Ricarda, a protected wetland in el Prat de Llobregat (Fig 1) in 2017, which were reared to adults on a diet of Frippak 3CD fish food at 25°C and >60% RH.

### Insecticide susceptibility assays

Insecticide susceptibility was tested using two different methods: CDC bottle bioassays and WHO tube tests.

#### CDC bottle bioassays

CDC bottle assays were performed following the guidelines of the Centers for Disease Control and Prevention [39]. Analytical standard lambda-cyhalothrin, deltamethrin, permethrin, pirimiphos-methyl, 4,4°-DDT and bendiocarb (Pestanal Analytical standard, Sigma-Aldrich) were dissolved in pure acetone to diagnostic concentrations (Table 1). All bottles (treatment and controls) were coated with insecticides on the same day of each test. To assess insecticide susceptibility levels, up to 25 two-to-six day old non-blood-fed female mosquitoes were introduced into each bottle, with four insecticide-coated and one control bottle in total (tests with *Ae*. *albopictus* from Figueras and Llorenç del Penedes contained half the numbers).

**Table 1:** Available information on the discriminating bioassay concentrations of insecticides for determining susceptibility of adult anopheline and culicine mosquitoes using WHO and CDC insecticide susceptibility tests for the insecticides used in our studies.

	Pyrethoids			Carbamates		Organophosphates	Organochlorines
	Deltamethrin	Lambda-cyhalothrin	Permethrin	Bendiocarb	Propoxur	Pirimiphos-methyl	DDT
**WHO tube assay (%)**							
Recommended dose *Anopheles* spp^a^	0.05	0.05	0.75	0.1	0.1	0.25	4
Recommended dose *Aedes* spp^a^	0.03^b^	0.03	0.25	-	-	0.21^c^	-
*Cx*. *pipiens tests 2015*^a^	0.05	-	-	-	0.1	0.25	4
**CDC bottle bio-assay** (**μg/bottle)**							
*Recommended dose Anopheles spp*^a^^,^^d^	12.5	12.5	21.5	12.5	-	20	100
*Recommended dose Aedes spp*^a^^,^^d^	10	10	15	12.5	-	-	75
*Ae*. *albopictus tests 2012–2013*^e^	1000	1000	3125	-	-	-	-
*Ae*. *albopictus tests 2016*	10	-	-	10^f^	-	20	75
*Ae*. *caspius tests 2017*	10	-	-	-	-	-	75
*Recommended dose Culex* spp	-	-	-	-	-	-	-
*Cx*. *pipiens tests 2012–2013*^e^	250	250	2500	-	-	-	-
*Cx*. *pipiens tests 2016*	10^g^/25^h^	-	-	10^f^	-	20^g^/40^h^	75
*Cx*. *pipiens tests 2017*	25^h^	-	-	25^h^	-	40^h^	200^h^

^a^ WHO guidelines [40]

^b^ tentative [40],

^c^ determined for *Anopheles*, tentative for *Aedes* [40] (we assume a typo, as the value for *Anopheles* is 0.25),

^d^ CDC guidelines [39],

^e^ WHO guidelines for *Culex quinquefasciatus* [41], converted to *μ*g/bottle

^f^ lower dose by error

^g^ value for *Aedes* used

^h^ twice the *Anopheles* dose, as recommended by Dr. William Brogdon (CDC, Atlanta, personal communication)

#### WHO tube test

WHO tube tests were performed according to the guidelines published by the WHO [40] using WHO-standard insecticide-treated papers (Table 1). For each insecticide tested, approximately 100 mosquitoes (4 replicates of 25) for the exposed group and 50 mosquitoes (2 replicates of 25) for the control group were kept in a holding tube lined with untreated paper. Next, they were exposed for 1 hour to insecticide-treated or control paper, respectively, using WHO-standard exposure tubes and procedures. After exposure, mosquitoes were transferred to paper holding cups and had *ad libitum* access to sugar water (10% household sugar solution).

#### Insecticide exposures

*Cx pipiens* mosquitoes collected from various areas within Catalonia in 2012–2014 were tested using the CDC bottle assay with lambda-cyhalothrin, deltamethrin and permethrin. *Cx pipiens* mosquitoes from El Prat de Llobregat in 2015 were exposed to deltamethrin, propoxur, pirimiphos-methyl and 4,4’-DDT using WHO tube test. The collected *Cx pipiens* mosquitoes from 2016–2017 were exposed to deltamethrin, pirimiphos-methyl, 4,4´-DDT and bendiocarb in the CDC bioassay (Table 1).

*Ae*. *albopictus* mosquitoes collected in 2012/2014 were exposed to lambda-cyhalothrin, deltamethrin and permethrin, and in 2016, these mosquitoes were tested with deltamethrin, pirimiphos-methyl, 4,4´-DDT and bendiocarb. All tests on *Ae*. *albopictus* mosquitoes were performed with CDC bottle assays. *Ae*. *caspius* mosquitoes from 2017 were exposed to DDT and deltamethrin in the CDC bottle assay.

#### Insecticide concentrations

Of note is that the diagnostic dosages for insecticide resistance are not clear for each mosquito species or indeed some genera. As there were no established diagnostic doses for *Culex* species in general, but see [41] for guidance on individual mosquito species for some insecticides, two sets of susceptibility tests were conducted in 2016. In the first test, *Cx*. *pipiens* were exposed to diagnostic doses of pirimiphos-methyl, DDT and deltamethrin concentrations as determined for *Aedes* species. In the second test, mosquitoes from the same population were exposed to twice the diagnostic dose for *Anopheles* species (personal communication Dr. William Brogdon, CDC, Atlanta). For bendiocarb 10μg/bottle was accidentally used opposed to the recommended diagnostic dose of 12.5μg/bottle. The different recommended concentrations known for each mosquito genera and the insecticide concentrations used in this study are summarized in Table 1.

### Data analysis

For the WHO tube bioassay mortality was assessed twenty-four hours post-exposure in each replicate. In the CDC bottle bioassay, susceptibility was determined by quantifying mortality at a diagnostic time in each bottle. The diagnostic time was 30 minutes for for lambda-cyhalothrin, deltamethrin, permethrin, pirimiphos-methyl, and bendiocarb and 45 minutes for DDT. A mosquito was identified as ´dead´ if she lacked the ability to stand on her feet at a gentle rotation of the bottle or tube. When control mortality was between 5 and 20%, mortality was corrected using Abbott’s formula [40], with negative values being rounded to zero (note that control mortality is shown in S1 Table for *Cx*. *pipiens*, S2 Table for *Ae*. *albopictus* and S3 Table for Ae. *caspius*).

Resistance to the insecticide tested was identified as per the current WHO guidelines [40]. Mosquito populations were classified as susceptible if mortality ranges from 98 to 100% 24*h* after insecticide exposure. Mosquito mortality between 90 and 97% was defined as suspected resistance but would require follow-up testing to confirm resistance (if resistance is again below 98%). If mortality is less than 90%, resistance is confirmed and -if at least 100 mosquitoes are tested- no additional bioassays are needed.

## Results

### Culex pipiens

Multiple biotypes have been detected in population from the following localities: Bellaterra (biotypes *pipiens* and *molestus*); Bellvis (biotypes *pipiens* and *molestus*); Gava’ (biotypes *pipiens* and *hybrid*); Santa Coloma de Cervelló (biotypes *pipiens* and *hybrid*), Vic (biotypes *pipiens*, *molestus* and *hybrid*). The population from Empuriabrava was characterized by the presence of a unique biotype: *Cx*. *pipiens pipiens* biotype *molestus*.

Resistance in *Cx*. *pipiens* to deltamethrin was observed in all six locations studied in Catalonia during 2012–2014 (Table 2). Resistance to lambda-cyhalothrin was seen in two of the six areas and suspected in the other three areas. Resistance to permethrin was detected in one of the six areas and suspected in two other areas. Mortality in response to deltamethrin exposure was particularly low in Bellvis (4%).

**Table 2:** Insecticide susceptibility of *Cx*. *pipiens* collected from various localities in Catalunya (Spain). Percentage indicates percent mortality (WHO tube tests: 24*h* following 1*h* exposure; CDC bottle assays: At discriminating exposure time of 30 min (45 min for DDT)); number between parentheses indicates the number of mosquitoes tested.

	Pyrethroids				Carbamates		Organochloride		Organophosphate	
	Permethrin	Deltamethrin		Lamda-cyhalthrin	Bendiocarb	Propoxur	DDT		Pirimiphos-methyl	
	CDC	WHO	CDC	CDC	CDC	WHO	WHO	CDC	WHO	CDC
2012–2014										
Bellaterra	97% (100)	-	80% (100)	100% (100)	-	-	-	-	-	-
Bellvis	81% (100)	-	4% (100)	55% (100)	-	-	-	-	-	-
Empuriabrava	95% (100)	-	62% (100)	97% (100)	-	-	-	-	-	-
Gavà	99% (100)	-	35% (100)	85% (100)	-	-	-	-	-	-
Santa Coloma de Cervelló	98% (100)	-	63% (100)	93% (100)	-	-	-	-	-	-
Vic	99% (100)	-	87% (100)	95% (100)	-	-	-	-	-	-
2015										
Torrelles de Llobregat	-	81.2% (101)	-	-	-	0% (91)	6.3% (96)	-	0% (98)	-
2016										
Torrelles de Llobregat	-	-	96.1% (85)^1^ 97.5% (91)^2^	-	-	-	-	78.2% (58)	-	0% (71)^3^ 0% (49)^4^
2017										
El Prat de Llobregat	-	-	96.2% (106)^2^	-	46.2% (104)	-	-	68.3% (101)	-	33.3% (90)

^1^10 μg/bottle;

^2^25 μg/bottle;

^3^20 μg/bottle;

^4^40 μg/bottle

Additional testing with *Cx*. *Pipiens* populations from the Barcelona area in 2015 showed this species was resistant to all four public health insecticides: deltamethrin (81% mortality), DDT (6%), pirimiphos-methyl (0%) and propoxur (0%) (Table 2). Subsequent tests (CDC bottle bioassay) confirmed resistance to the three chemical classes tested (pyrethroid deltamethrin: 96% [2016] and 96% [2017] mortality; organophospate pirimiphos-methyl: 0% [2016] and 33% [2017]; organochloride DDT: 78% [2016] and 68% [2017]). Deltamethrin and pirimiphos-methyl exposures were repeated in 2016 with a higher diagnostic dose (twice the dose for *Anopheles*, Table 1), which showed clear resistance to pirimiphos-methyl (0% mortality) and suspected resistance to deltamethrin (97.5%) (Table 2). Resistance to bendiocarb (46% mortality) was observed in 2017.

### Aedes albopictus

No resistance to pyrethroids was detected in the *Ae*. *albopictus* populations tested throughout Catalonia during 2012–2014 (Table 3). Additional tests in 2016 with mosquitoes from the Barcelona area showed that *Ae*. *albopictus* populations was fully susceptible to deltamethrin, bendiocarb and DDT, but showed levels of resistance to pirimiphos-methyl (86.52% mortality) in one out of the two tests performed.

**Table 3:** Insecticide susceptibility of *Ae*. *albopictus* collected from various localities in Catalunya (Spain). Percentage indicates percent mortality at discriminating exposure time of 30 min (45 min for DDT); number between parentheses indicates the number of mosquitoes tested.

	Pyrethroids			Carbamates	Organochloride	Organophosphate
	Permethrin	Deltamethrin	Lamda-cyhalthrin	Bendiocarb	DDT	Pirimiphos-methyl
	CDC	CDC	CDC	CDC	CDC	CDC
2012–2014						
Constantí	100% (100)	99% (100)	100% (100)	-	-	-
El Prat	100% (100)	99% (100)	100% (100)	-	-	-
Figueras	100% (50)	100% (50)	100% (50)	-	-	-
Lloret de Mar	100% (100)	100% (100)	100% (100)	-	-	-
Llorenç del Penedes	100% (50)	100% (50)	100% (50)	-	-	-
Mont-Roig del Camp	100% (100)	98% (100)	100% (100)	-	-	-
Sant Julià de Ramis	100% (100)	100% (100)	100% (100)	-	-	-
2016						
Cornella de Llobregat	-	100% (77)	-	100% (88)	100% (86)	86.5% (88)
El Prat de Llobregat	-	100% (82)	-	-	-	98.5% (86)

### Aedes caspius

*Ae*. *caspius* (collected from the Barcelona area in 2017) was fully susceptible to DDT and deltamethrin (both 100% mortality, other chemical classes not tested) (Table 4).

**Table 4:** Insecticide susceptibility of *Ae*. *caspius* collected from Baix Llobregat (Barcelona, Spain) in 2017. Percentage indicates percent mortality at discriminating exposure time of 30 min (45 min for DDT); number between parentheses indicates the number of mosquitoes tested.

	Pyrethroids	Organochloride
	Deltamethrin	DDT
La Ricarda	100% (85)	100% (43)

## Discussion

Here we provide evidence that several *Cx*. *pipiens* populations from Catalonia and Barcelona, which are potential vectors of arboviruses like WNV, USUV and RVFV [26, 27, 42] are resistant to some of the pyrethroid insecticides evaluated. We observed spatial variation in resistance phenotype, with high levels of resistance in Bellvis and to a lesser extent in Gava. One explanation for this observation could bethe extensive use of pyrethroids in the agricultural activity in the past and at present. In contrast, no large and repetitive adult and larval control have been applied in the area with these products nor in the past nor at present. Since the 1980’s, mosquito control in the area has been conducted with larvicides, formerly Temephos and since 1992 with Bti. No pyrethroids have been used as larvicides. Few adulticides have been conducted with Malathion, except for occasional adulticiding with pyrethroids. The only possible continuous application of adulticides using this family of insecticides originates from domestic insecticide devices that are largely used by public in general. Moreover, the *Cx*. *pipiens* populations from the Barcelona area are resistant to insecticides belonging to all four classes of public health insecticides available: Resistance is clearly demonstrated for DDT (organochloride), pirimiphos-methyl (organophosphate), propoxur (carbamate) and various pyrethroids. Again, one explanation is the widely use of DDT in agriculture and public health in the past in all Spain until 1977 when it was banned and the possible illegal use has been certainly almost inexistent. On the other hand, insecticide resistant genes may have migrated from other parts of Europe and/or the world, as extensive and long-distance migration of genes has been observed in *Cx*. *pipiens* [43].

In contrast, *Ae*. *albopictus*, a potential vector of DENV, ZIKV, CHIKV and USUV [42] was susceptible to all four classes of public health insecticides across different years and methodologies used. However, a single observation suggested some level of resistance to the organophosphate pirimiphos-methyl, which needs to be confirmed. These findings are similar to those observed in other European *Ae*. *albopictus* populations, such as in Switzerland [44]. However, pyrethroid resistance seems to be emerging in European *Ae*. *albopictus* populations, as observed in Italy [45] and in Spain, with possible resistance to cypermethrin in Barcelona [46], and the kdr mutation (V1016G) has been recently reported in Italy [47].

*Ae*. *albopictus* was first detected in Spain in 2004 close to the Llobregat study area [23]. Although it appears to be spreading across Spain, recent genetic analysis revealed that present populations of *Ae*. *albopictus* are likely linked to multiple independent introduction events into Spain [48]. The absence of observed resistance to all four classes of insecticides in the populations tested suggests that these incoming populations may not have experienced significant insecticide pressure before being introduced in Spain (either through vector control interventions, or via agricultural applications).

*Ae*. *caspius* was fully susceptible to deltamethrin, similar to observations in Greece [49], and to DDT. Its observed susceptibility is likely linked with its ecological niche: it is constrained to swampy natural areas, environments that are not human-made and where typically few pesticides are applied. This contrasts with *Cx*. *pipiens* habitats that are often man-made and have been shown to contain large insecticide quantities in the past [50].

It is important to keep in mind that obtaining meaningful results for some chemical classes, particular insecticides and mosquito species is difficult. There is no agreement yet on the discriminating concentrations for *Aedes* in the WHO tube bioassays [51] and data are missing for several chemical classes and insecticides. The situation for *Culex* vectors is worse: there are no guidelines on diagnostic concentrations apart from some scarce information on some species and few insecticides [41]. Regarding the CDC bottle bioassay [39], the diagnostic doses and times have been established for most insecticides, but not for pirimiphos-methyl (*Aedes*), and there is again no information for *Culex* species. As such, the observed resistance in *Cx*. *pipiens* may not be ‘true’ resistance, but simply an artefact of the doses and/or diagnostic time used. Preferably, dose-response curves are generated to identify the discriminating dose for each species and each insecticide, after which longitudinal susceptibility studies starting prior to insecticide use are implemented to observe changes over time. However, this is rarely practiced and indeed not the case for our study area.

In addition, it is key to consider that the outcomes presented pertain to standardized laboratory bio-assays, following existing guidelines and using suggested diagnostic dosages. Susceptibility in such conditions do not necessarily reflect susceptibility in programmatically applied interventions in field situations. Several other factors, not considered in these standard susceptibility tests, have been shown to impact insecticide toxicity, such as exposure temperature [52], variations in the larval environment [53], mosquito age [54], (multiple) blood feeding events [55] and infections with pathogens [56]. Moreover, actual doses and contact times with the insecticide following space spraying may vary in the field and differ from those tested in the lab. This will likely impact the efficacy of fogging campaigns and -as a consequence- disease transmission potential. Furthermore, mosquitoes that do survive an insecticide exposure may still die within a few days, reducing their transmission potential compared to unexposed mosquitoes, a phenomenon referred to as delayed mortality [57]. In other words, the classification ‘resistant’ does not necessarily imply an insecticide will not be effective in the field. This ties in with the much larger debate on the impact (or not) of insecticide resistance on disease transmission [58–60].

## Conclusions

At the moment insecticides remain one of the corner stones of arboviral disease outbreak control and prevention. The results presented here indicate that *Ae*. *albopictus* populations across Catalonia can be controlled by space spraying with pyrethroids and maybe -at least in the Barcelona area- with organophosphates, the two chemical classes approved for space spraying by WHO [61]. For residual spray applications (of e.g. mosquito resting areas inside homes or cattle stables/sheds) the carbamate class can be used (again only confirmed for Barcelona). Having said that, the number of chemical classes approved for adulticiding by the European Union is limited, with pyrethroids being exclusively recommended in local and national guidelines [62]. Unfortunately, the situation for *Cx*. *pipiens* appears worse (but note the discussion on appropriate diagnostic doses/times) as resistance to all four classes of public health insecticides has been observed. As we rely heavily on a very limited choice of chemicals (pyrethroids are currently the first line chemical class for adulticiding in response to outbreaks of any arboviral disease), proper resistance management strategies should be in place [51] for any vector species that is targeted. This necessitates continuous entomological surveillance to monitor vector population dynamics and their insecticide susceptibility status [63].

## Supporting information

S1 Table Control mortality during insecticide susceptibility testing of *Cx*. *pipiens* collected from various localities in Catalunya (Spain). (DOCX)

S2 Table Control mortality during insecticide susceptibility testing of *Ae*. *albopictus* collected from various localities in Catalunya (Spain). (DOCX)

S3 Table Control mortality during insecticide susceptibility testing of *Ae*. *caspius* collected from Baix Llobregat (Barcelona, Spain) in 2017. (DOCX)
