# Correction: Phenotypic insecticide resistance in arbovirus mosquito vectors in Catalonia and its capital Barcelona (Spain)

**DOI:** 10.1371/journal.pone.0229122

**Published:** 2020-02-06

**Authors:** Krijn Paaijmans, Marco Brustolin, Carles Aranda, Roger Eritja, Sandra Talavera, Nonito Pagès, Silvie Huijben

The second author's name is spelled incorrectly. The correct name is: Marco Brustolin. The correct citation is: Paaijmans K, Brustolin M, Aranda C, Eritja R, Talavera S, Pagès N, et al. (2019) Phenotypic insecticide resistance in arbovirus mosquito vectors in Catalonia and its capital Barcelona (Spain). PLoS ONE 14(7): e0217860. https://doi.org/10.1371/journal.pone.0217860
