# Supplementary material for: Phenotypic insecticide resistance in arbovirus mosquito vectors in Catalonia and its capital Barcelona (Spain)
Source: PLoS One. 2019 Jul 5;14(7):e0217860. doi: 10.1371/journal.pone.0217860 (PMC6611561; doi:10.1371/journal.pone.0217860)
Supplement: S1 Table — (DOCX) [file pone.0217860.s001.docx]

**S1 Table**. Control mortality during insecticide susceptibility testing of *Cx. pipiens* collected from various localities in Catalunya (Spain). Percentage indicates percent mortality (WHO tube tests: 24*h* following 1*h* exposure; CDC bottle assays: at discriminating exposure time of 30 min (45 min for DDT)); number between parentheses indicates the number of mosquitoes tested.

|  | Pyrethroids | | | | Carbamates | | Organochloride | | Organophosphate | |
| --- | --- | --- | --- | --- | --- | --- | --- | --- | --- | --- |
|  | Permethrin | Deltamethrin | | Lamda-cyhalthrin | Bendiocarb | Propoxur | DDT | | Pirimiphos-methyl | |
|  | CDC^1^ | WHO | CDC^1^ | CDC^1^ | CDC | WHO | WHO | CDC | WHO | CDC |
| 2012-2014 |  |  |  |  |  |  |  |  |  |  |
| Bellaterra | 2% (100) | - | 2% (100) | 2% (100) | - | - | - | - | - | - |
| Bellvis | 1% (100) | - | 1% (100) | 1% (100) | - | - | - | - | - | - |
| Empuriabrava | 0% (100) | - | 0% (100) | 0% (100) | - | - | - | - | - | - |
| Gavà | 1% (100) | - | 1% (100) | 1% (100) | - | - | - | - | - | - |
| Santa Coloma de Cervelló | 0% (100) | - | 0% (100) | 0% (100) | - | - | - | - | - | - |
| Vic | 0% (100) | - | 0% (100) | 0% (100) | - | - | - | - | - | - |
| 2015 |  |  |  |  |  |  |  |  |  |  |
| Torrelles de Llobregat | - | 0% (50) | - | - | - | 6% (49)^2^ | 0% (49) | - | 6% (49)^2^ | - |
| 2016 |  |  |  |  |  |  |  |  |  |  |
| Torrelles de Llobregat | - | - | 0% (17)^3^  0% (15)^4^ | - | - | - | - | 0% (10) | - | 0% (12)^5^  0% (14)^6^ |
| 2017 |  |  |  |  |  |  |  |  |  |  |
| El Prat de Llobregat | - | - | 0% (23)^4^ | - | 4% (25) | - | - | 0% (28) | - | 0% (28) |

^1,2^Shared control; ^3^10 *µ*g/bottle; ^4^25 *µ*g/bottle; ^5^20 *µ*g/bottle; ^6^40 *µ*g/bottle
