# Supplementary material for: Phenotypic insecticide resistance in arbovirus mosquito vectors in Catalonia and its capital Barcelona (Spain)
Source: PLoS One. 2019 Jul 5;14(7):e0217860. doi: 10.1371/journal.pone.0217860 (PMC6611561; doi:10.1371/journal.pone.0217860)
Supplement: S2 Table — (DOCX) [file pone.0217860.s002.docx]

**S2 Table**. Control mortality during insecticide susceptibility testing of *Ae. albopictus* collected from various localities in Catalunya (Spain). Percentage indicates percent mortality at discriminating exposure time of 30 min (45 min for DDT); number between parentheses indicates the number of mosquitoes tested.

|  | Pyrethroids | | | Carbamates | Organochloride | Organophosphate | |
| --- | --- | --- | --- | --- | --- | --- | --- |
|  | Permethrin | Deltamethrin | Lamda-cyhalthrin | Bendiocarb | DDT | | Pirimiphos-methyl |
|  | CDC^1^ | CDC^1^ | CDC^1^ | CDC | CDC | | CDC |
| 2012-2014 |  |  |  |  |  | |  |
| Constantí | 0% (100) | 0% (100) | 0% (100) | - | - | | - |
| El Prat | 1% (100) | 1% (100) | 1% (100) | - | - | | - |
| Figueras | 0% (50) | 0% (50) | 0% (50) | - | - | | - |
| Lloret de Mar | 0% (100) | 0% (100) | 0% (100) | - | - | | - |
| Llorenç del Penedes | 0% (50) | 0% (50) | 0% (50) | - | - | | - |
| Mont-Roig del Camp | 0% (100) | 0% (100) | 0% (100) | - | - | | - |
| Sant Julià de Ramis | 0% (100) | 0% (100) | 0% (100) | - | - | | - |
| 2016 |  |  |  |  |  | |  |
| Cornella de Llobregat | - | 0% (20) | - | 0% (22) | 0% (13) | | 0% (19) |
| El Prat de Llobregat | - | 0% (14) | - | - | - | | 0% (16) |

^1^Shared control
