# Supplementary material for: Phenotypic insecticide resistance in arbovirus mosquito vectors in Catalonia and its capital Barcelona (Spain)
Source: PLoS One. 2019 Jul 5;14(7):e0217860. doi: 10.1371/journal.pone.0217860 (PMC6611561; doi:10.1371/journal.pone.0217860)
Supplement: S3 Table — (DOCX) [file pone.0217860.s003.docx]

**S3 Table**. Control mortality during insecticide susceptibility testing of *Ae. caspius* collected from Baix Llobregat (Barcelona, Spain) in 2017. Percentage indicates percent mortality at discriminating exposure time of 30 min (45 min for DDT); number between parentheses indicates the number of mosquitoes tested.

|  | Pyrethroids | Organochloride |
| --- | --- | --- |
|  | Deltamethrin | DDT |
| La Ricarda | 0% (11) | 0% (14) |
